# Unveiling the dynamics of ultra high velocity droplet impact on solid surfaces

**DOI:** 10.1038/s41598-022-11188-7

**Published:** 2022-05-06

**Authors:** Giovanni Tretola, Konstantina Vogiatzaki

**Affiliations:** Department of Engineering, King’s College London, Strand, London, WC2R 2ND UK

**Keywords:** Aerospace engineering, Mechanical engineering

## Abstract

The impact of a liquid droplet onto a solid surface is a phenomenon present in a wide range of natural processes and technological applications. In this study, we focus on impact conditions characterised by ultra high velocities (up to 500 m/s), to investigate—for the first time—how the impact dynamics change when the compressibility of the liquid in the droplet is no longer negligible. A water droplet impacting a dry substrate at four different velocities, from 50 to 500 m/s, is simulated. Such conditions are particularly relevant to aviation as well as industrial gas turbine engine risk management. Thus, numerical investigations as the one we present here provide a powerful tool to analyse the process. We find that increasing the impact velocity changes the flow field within and outside the droplet the moment that the compressibility can no longer be neglected, with the rise of pressure fronts in both regions. Increasing the impact velocity, the compressibility affects also the lamella formed and changes its ejection velocity observed over time (and thus the wetting behaviour) when the region shift from incompressible to compressible. Moreover, it is found that the maximum pressure observed at the wall during the impact is located at the corner of the impact, where the lamella is ejected, not in the centre, and it is influenced by the initial velocity. To predict the maximum pressure experienced by the surface during the high velocity impact, we propose a correlation based on the initial Weber and Reynolds number of the droplet. The complexity and the scales of the dynamics involved in the ultra-high velocity impact is limiting the experimental and analytical studies. To the best of our knowledge there are no experimental data currently available at such conditions. In this study, through numerical simulations, new insights about the impact dynamics at such conditions are provided.

## Introduction

The impact of a liquid droplet onto a solid surface is a phenomenon present both in nature and in technological applications, such as ink-jet printing, turbine operation, vehicle soiling, spray cooling and aircraft icing. Various complex physical phenomena occur during the impact including spreading, fingering, air entrapment, coalescence, shedding, solidification, bouncing, and splashing. Several reviews exist in the literature that classify these different impact outcomes^1–3^ based on the fluid properties, the surface properties and the impact velocity. Despite the large number of studies of droplet impact, the interplay of all these factors is still far from being fully understood in industrially relevant high speed impact cases. One of the areas that is less investigated is what happens inside the droplets during impact and the role of the surrounding gas. For example as a droplet impacts with high velocity (up to 500 m/s) onto a surface, compression waves can be created and propagate in the droplet, resulting in local density variations which in turn might rapture the interface and give rise to jets^4,5^. Also the surrounding gas (based on the work of Riboux & Gordillo^6^) can affect the impact as for example in the case of splashing that is attributed to aerodynamic lift force on the spreading lamella.

The outcome of the droplet impact has been categorised in different regimes based on non-dimensional parameters such as the Weber (*We*) and the Reynolds (*Re*) number for cases involving dry^7^, heated^8^, moving^9^, inclined^10^ or hydrophobic^11^ surfaces. Most of the models and scales to describe the different phenomena related to the droplet impact have been developed on experiments at low or moderate *We* and *Re* numbers. Examples of studies at the lower/moderate velocity regime include the work of Mehdizadeh et al.^12^ that investigated water droplet impingement with impact velocities from 10 to $50 ~\text{ m/s }$, to analyse the droplet spreading and fingering formation. They proposed a model, based on linear Rayleigh-Taylor instability theory to predict the wavelength of the fastest growing perturbation around a spreading droplet. Wang et al.^13^ investigated the impact behavior of normal and oblique impacts on superhydrophobic surfaces at very low Weber ($We <1$), including also the effect of wettability. Wang et al.^14^ investigated experimentally the head-on collisions of a liquid droplet at low Weber ($We \ge 5$) with another of the same fluid resting on a solid hydrophobic substrate, proposing a theoretical model to estimate the contact time for the rebound cases. Wilderman et al.^15^ proposed a model (based on energy balance) to predict droplet spreading when impacting both lubricated and no-slip surfaces. The authors focused on studying droplet impacting with *We* ranging from 0.3 to 300. Huang and Chen^16^ investigated experimentally and numerically the impact of droplet at velocities up to $\approx 4$ m/s, giving estimations of the relative quantities in the energetic analysis, thus, improving the traditional energetic model. Guo et al.^17^ experimentally and numerically investigated the spreading of impinging droplets of different viscosities and sizes on superhydrophobic surfaces, with impacting velocities up to $3~\text{ m/s }$, showing an higher influence of the impact velocity and liquid viscosity over the initial diameter. Visser et al.^18^ analysed higher velocity impact (up to $100 ~\text{ m/s }$) of water microdroplets investigating if splashing occurs. They showed that in this case splashing is not observed and that the droplets always gently spread over the surface.

Challenges such as wide scale ranges involved during the impact, from the droplet diameter to the lamella, ranging from millimetres to microns, and experimental limitations to access the interior of the droplet during the impact, have limited the number of experimental studies at high velocity impacts that are of industrial interest. For example one of the few studies in the area is the one recently published by Burzynski et al.^19^ that investigated impact at high *We* and *Re* numbers in order to quantify the impact outcome when liquid droplets hit dry rigid surfaces. Their focus was on the secondary droplets produced. They showed that the *Re* affects the splashing more than the *We* does. Furthermore, they linked the theory proposed by Riboux & Gordillo^20^ to estimate the entire outcome of the splash, such as size, velocity, angle and total ejected volume of the secondary droplets. Aboud et al.^21^ experimentally analysed the oblique drop impacts at high *We* (up to $27 ~\text{ m/s }$, $We > 9000$) on different substrates, showing the influence of the substrate on the splashing conditions. They showed also the influence of the surface roughness on the symmetric nature of the splashing and found a new behaviour in highly oblique impacts on superhydrophobic surfaces. In both works no visualisation of the interior of the droplet was provided.

Numerical investigations are a necessary complementary tool to explore these impact conditions and can provide valuable insight to areas like the droplet interior that experiments face limitations. However, this direction is also challenging due to the complex numerics involved and thus only a limited number of studies exist Cimpeanu & Papageorgiou^22^ conducted pre-impact deformation studies through Direct Numerical Simulations (DNS), with high velocities impact conditions ($8000< Re <$ 80,000 and 10,000 < *We* < 90,000), considering non-stationary air flow to quantify pre–impact, spreading and splashing dynamics. Marzbali et al.^23^ quantified the impact pressure of liquid droplets at velocities up to $500~\text{ m/s }$, through numerical simulations, on rigid solid substrates and liquid films.

In this study we analyse, for the first time, how the high velocity droplet impact dynamics change when moving from an incompressible to a compressible impact regime, linking internal pressure waves to the macroscopic droplet morphology, impact dynamics, and lamella spreading. As a relevant case, the impact of a water droplet hitting a solid surface at different velocities is simulated, characterised by *We* and *Re* up to $\approx$ 5,00,000 and $\approx$ 10,00,000 respectively. First, the model used is briefly presented, followed by the set up of the cases investigated. The results of the simulated impact dynamic are then observed, with a focus on the influence of the initial impact velocity. The droplet morphology during the impact and its dynamic is discussed, both qualitatively and quantitatively. Then, the gaseous flow field is analysed, followed by the examination on the lamella ejected.

## Methods

The details of the numerical methodology adopted in this study are presented in the supplementary material. Here only the main points of the methodology and the set-up employed are included. Our suggested framework is designed for simulations of compressible and immiscible fluids. The interface tracking of the two-phase flow is performed with the Volume of Fluid (VoF) method as basis using the Open Source code OpenFOAM^24^. The native solver *compressibleInterFOAM* was used as starting point and modifications were made to account for the interface treatment at high velocity impact conditions. More details about the implementation into the method and the influence of these modifications, along with the validation of the implemented solver, can be found in Tretola and Vogiatzaki^25^.

**Figure 1:**
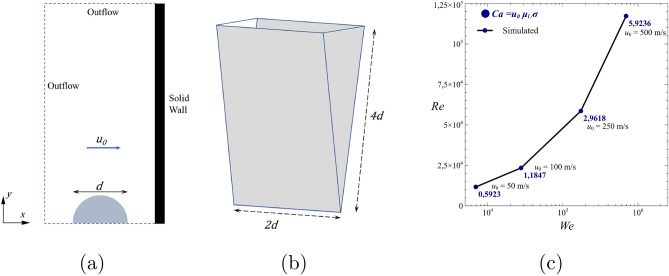
(**a**) Initial configuration and boundary conditions; (**b**) computational domain; (**c**) Operating point investigated on a *We*-*Re* map.

The impact conditions are defined in terms of *We* and *Re*. The first non dimensional number represents the ratio between the fluid inertia and the surface tension force and is defined as $We = \rho _l u_{0}^2 d_{0} /\sigma$, where $u_{0}$ is the initial impact velocity, $d_{0}$ the initial droplet diameter, $\rho _l$ the liquid density and $\sigma$ the surface tension. The Reynolds number represents the ratio of inertial forces to viscous forces, is defined as $Re = \rho _l u_{0} d_{0} /\mu _l$, where $\mu _l$ is the liquid dynamic viscosity. To investigate the impact at very high *We* and *Re*, a water droplet of diameter $d = 200~{\mu }{\mathrm{m}}$ at standard conditions is simulated at four different impact velocities $u_{0}$ equal to 50, 100, 250 and $500{~\text{ m/s }}$. The corresponding *We* and *Re* are reported in Fig. 1c. We remind here that the complexity of the droplet impact at such conditions limits the experimental studies and thus detailed experimental data of high-speed impact, with velocities over $100~\text{ m/s }$, of single droplets are not available in literature. It is worth noting that impacts at velocities over $100~\text{ m/s }$ have timescales of the order of nanoseconds, which consists into a constraint about the temporal resolution of the equipment.

The computational domain, illustrated in Fig. 1, consists of a 3D wedge geometry with one cell thickness. Its width is $2 \ d$, and the height of the domain is $4 \ d$. The gravitational force is exerted in the same direction as the droplet impingement. The computational domain consists of air and water phases, with temperature and pressure of $T = 300~\text{ K }$ and $p = 100~\text{ kPa }$ for both phases. The outflow boundary condition is applied to all fluid boundaries except for the substrate surface, while no-slip, no-penetration, and adiabatic conditions are imposed on the fluid-solid interface as in Marzbali et al.^23^. At the investigated conditions, characterised by very high Weber number ($>10^4$), the droplet impact will evolve rapidly, with timescales of the order of nanoseconds. Thus, contact angle boundary conditions are not explicitly defined and a zero gradient boundary condition for the water volume fraction on the wall is imposed, as commonly done in literature for these cases. Following Marzbali et al.^23^, initially, the fluid domain is filled with air. At the beginning of each simulation, the droplet is patched in the fluid domain according to the impact conditions.

Regarding the equation of state, the ideal gas law is applied for the gaseous phase

1 $$\begin{aligned} \rho _g = \frac{p}{RT} \end{aligned}$$

where *R* is the specific gas constant equal to 287 J/kg K for air. For the liquid phase, the power law equation of state proposed by Tait is employed:

2 $$\begin{aligned} \rho _l = \rho _{l,0} \left( \frac{p + B}{p_a + B} \right) ^{1/A} \end{aligned}$$

where $\rho _{l,0}$ = 1000 kg/m$^3$ is the density of the water at ambient condition, $B = 300$ MPa, $p_a = 0.1$ MPa and $A = 7.415$.

The results are normalised to have a proper comparison varying the impact velocity. The length and velocity scales used to normalise are the initial droplet diameter and velocity, respectively $d_0$ and $u_0$. To normalise the pressure, the hydraulic shock or water hammer pressure is adopted, which is the pressure spike caused by a sudden variation of the liquid flow rate. It is defined as $p_{wh} = (\rho u_0 a)$, where $\rho$ and *a* are the water density and speed of sound, respectively. The definitions of the different normalisations are summarised in Table 1.

**Table 1:** Definition of normalised variables.

Time	Velocity	Pressure	Pressure Gradient
$t^* = t\ u_{0}/d$	$u^* = u/u_0$	$p^* = p/(\rho u_0 a)= p/p_{wh}$	$\nabla p^* = \nabla p / ( p_{wh}/d)$

## Results

### Internal droplet morphology

**Figure 2:**
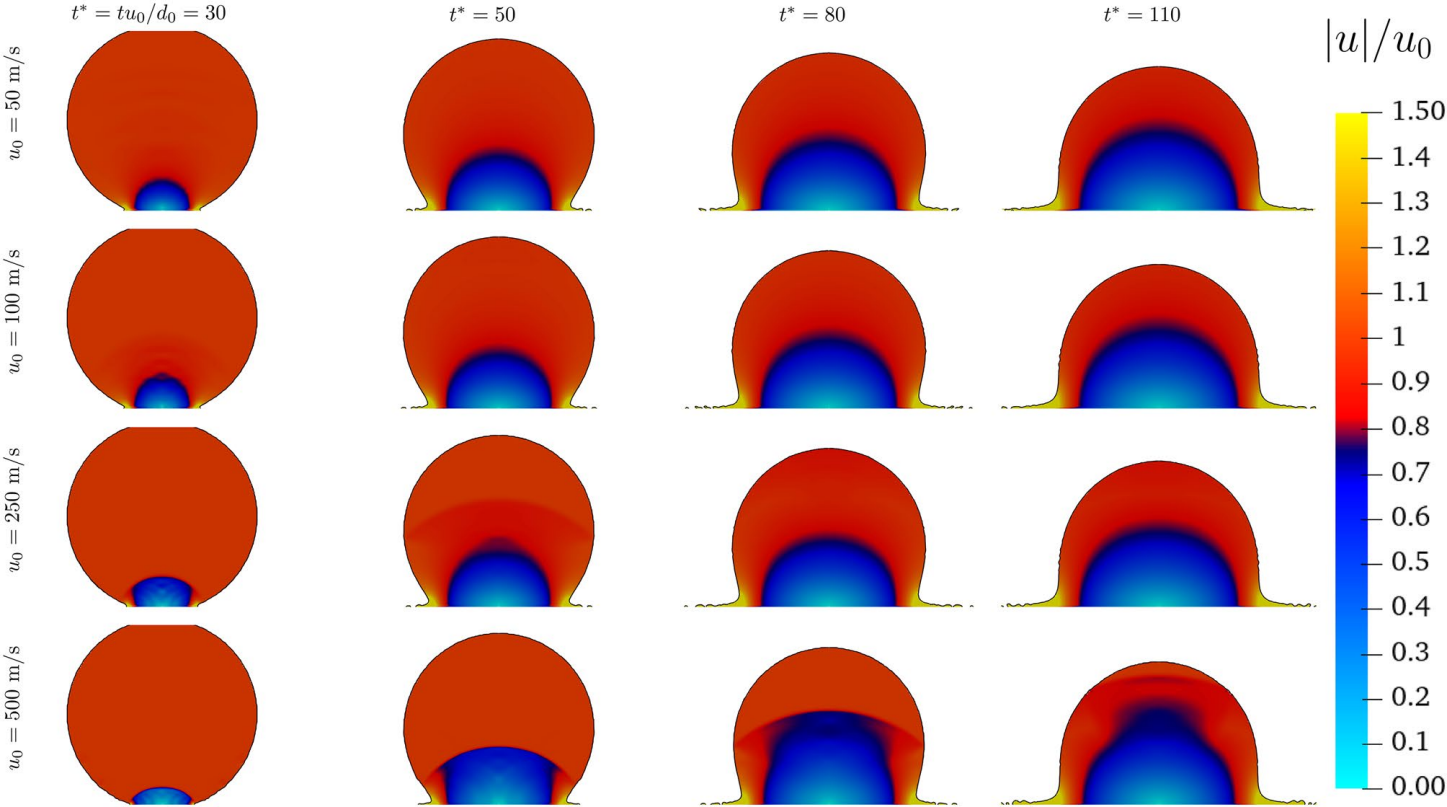
Velocity distribution within the droplet for different impact velocities at different $t^*$.

**Figure 3:**
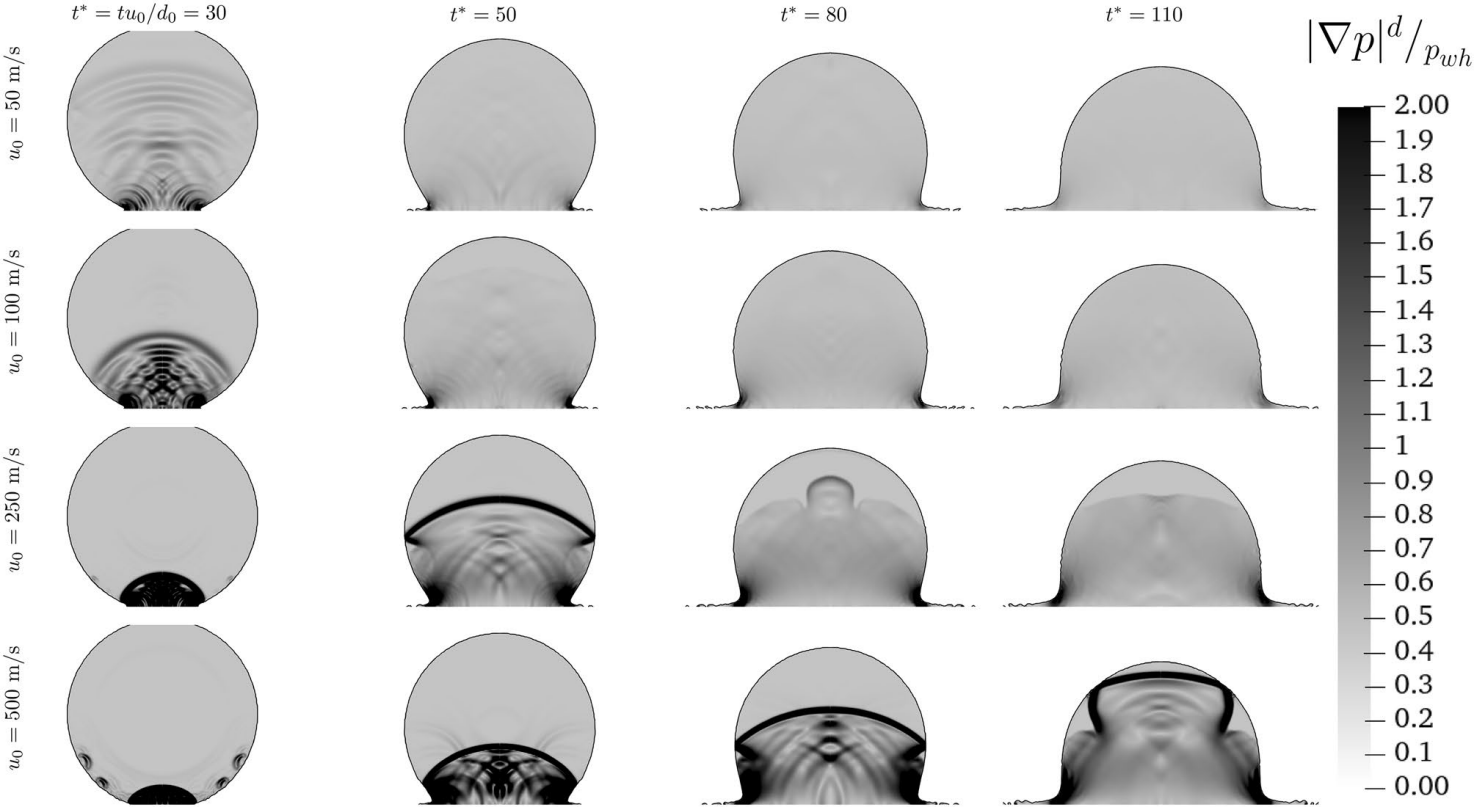
Dimensionless pressure distribution within the droplet for different impact velocities at different $t^*$.

Figure 2 shows the evolution of the velocity magnitude, made dimensionless by the impact velocity $u_{0}$, for the different velocities at the same dimensionless instants $t^*$. It is interesting to observe that in terms of normalised velocity, the pattern within the droplet is similar for the different $u_{0}$. A stagnation point forms at the impact points and as the impact dynamics evolve, it extends within the droplet following the pressure gradient observed in Fig. 3. For the velocity, a clear discontinuity is observed only for $u_{0} = 500~\text{ m/s }$, with a normalised velocity jump from 1 to 0.5. For $u_{0} = 250 ~\text{ m/s }$, the pressure front observed results in a smoother velocity jump (from 1 to 0.9) at $t^* =50$. From the impact a lamella is formed, characterised by a velocity $u \ge 1.5 u_{0}$ for all the cases investigated.

As mentioned above, the discontinuities observed in the velocity fields (Fig. 2) are linked to the pressure gradient. The pressure gradient evolution is shown in Fig. 3. For all the cases, the impact generates pressure waves that propagate from the impact point towards the top of the droplet. For low velocities ( $u_0 = 50$ and $100~\text{ m/s }$) though these waves quickly dissipate. As the initial impact velocity is increased, a stronger discontinuity within the droplet is observed due to the rise of the compressibility, which in turn intensifies the presence of travelling propagation fronts observed.

**Figure 4:**
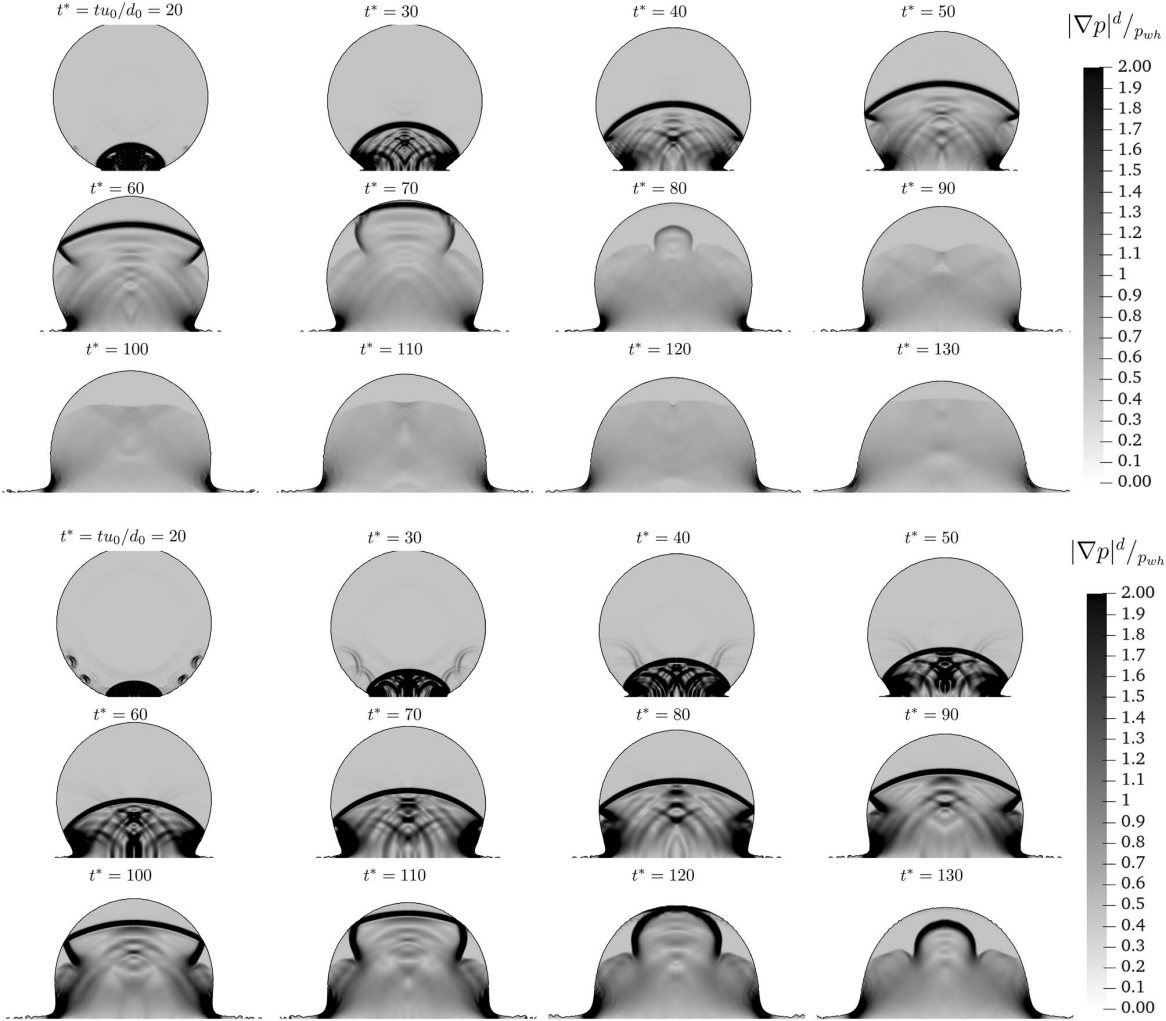
Dimensionless pressure gradient distribution within the droplet for $u_0 = 250~\text{ m/s }$ (*top*) and $u_0 = 500 ~\text{ m/s }$ (*bottom*) over time.

Increasing the impact velocity, also induces a second pressure front, with $|\nabla p| \ge 2 p_{wh}/d$, which starts to rise within the droplet from the impact point in a direction normal to the wall. As the time progresses the compressed liquid region increases. The evolution of the normal to the wall pressure front presents differences when the velocity is increased from $u_0 = 250$ to $500~\text{ m/s }$. To have a better understanding of the evolution of these travel fronts and to highlight their role, in Fig. 4 we demonstrate—for the higher velocity cases—the evolution of these fronts for more instants (in comparison to Fig. 3). For $u_{0} = 500~\text{ m/s }$, since the first instant of the impact, small waves are formed from the side of the droplet. These waves then travel along the main propagation front. The presence of these waves slows the main propagation front, which travel across the droplet slower than the $u_0 = 250 ~\text{ m/s }$ case. Once the front reaches the top of the droplet, it is dissipated for the $u_0 = 250 ~\text{ m/s }$ case, while it is reflected back for the $u_{0} = 500 ~\text{ m/s }$ case.

### Impact dynamics

**Figure 5:**
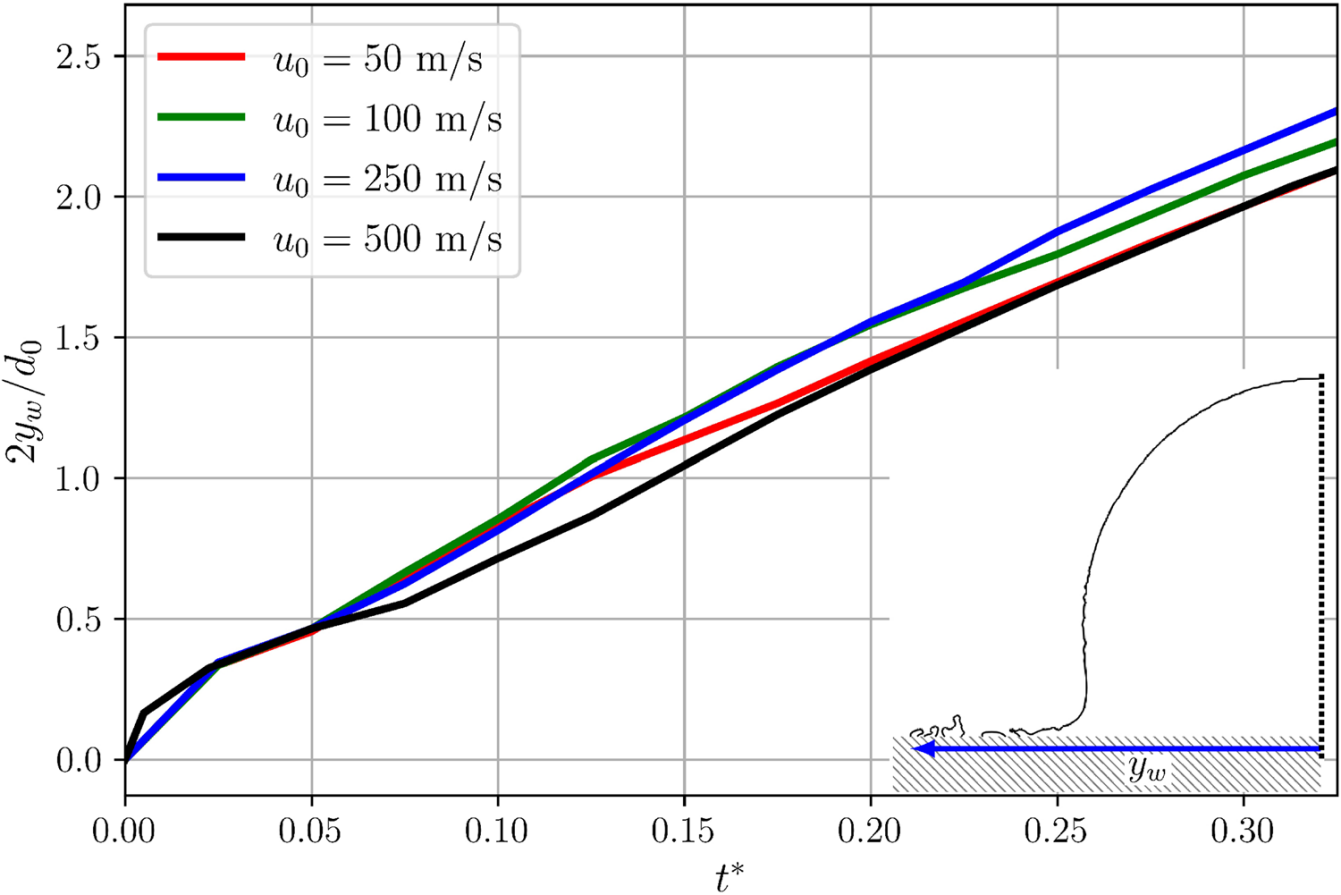
Trend over time, at different velocities, of spreading $y_w$ for the different cases, with its schematisation.

Figure 5 shows the distribution over time of the droplet spreading at the wall $y_w$, for the different impact velocities, normalised by the initial diameter. It is interesting to observe that the spreading stage is identical for all the cases regardless of the very different initial velocities. It is worth recalling that time is normalised as $t^* = t u_{0}/d$, which means that the non-dimensional dynamics are the same for the different velocities but not at the exact same time in absolute terms indicating a similarity of the event but with different rates. The similar spreading for the different $u_{0}$ is consistent with what observed experimentally in literature for $We \ge 3000$^26^.

**Figure 6:**
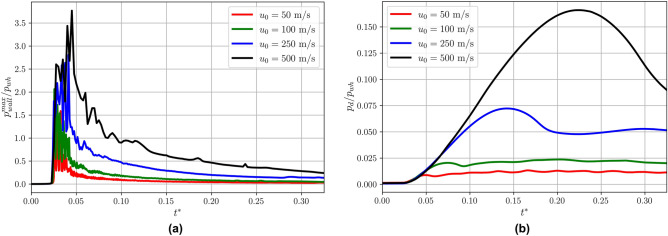
Distribution over time for different $u_{0}$ of: (**a**) Maximum pressure at the wall $p^{max}_{wall}$; (**b**) Droplet pressure $p_{d}$.

Figure 6 shows, the distribution over time of the maximum pressure at the wall $p^{max}_{wall}$, for the different impact conditions normalised by the water hammer pressure $p_{wh} = \rho _l u_0 a$ (a) and the droplet pressure $p_{d}$ over time (b). The droplet pressure $p_d$ is defined as

3 $$\begin{aligned} p_d = \frac{\int _{V} \alpha p dx }{\int _V \alpha dx } \end{aligned}$$

For all the cases, the impact of the droplets results in a water hammer. Several pressure peaks, higher than the water hammer pressure $p_{wh}$, are observed in the earlier stage of the impact ($t^*\in [0.02:0.05]$). These pressure peaks increase with $u_{0}$. Looking at the droplet pressure (Fig. 6b), increasing $u_{0}$, $p_{d}$ increases with the maximum value reached later. As it is shown by $p_{d}$ profiles, the droplet pressure rises at the early stage following the same profile, until $t^* = 0.025$. Then, for the first two impact cases $u_{0} = 50~\text{ m/s }$ and $100~\text{ m/s }$, after the maximum is reached, the pressure $p_{d}$ stabilises at a slightly lower value, $0.01 p_{wh}$ and $0.02 p_{wh}$ respectively. The different trend observed at higher $u_{0}$ is due to the pressure front travelling within the droplet (see Fig. 3). When the compressibility becomes relevant, at higher velocities the pressure waves travel as a discontinuity in the droplet increasing the pressure within the droplet and at the wall.

**Figure 7:**
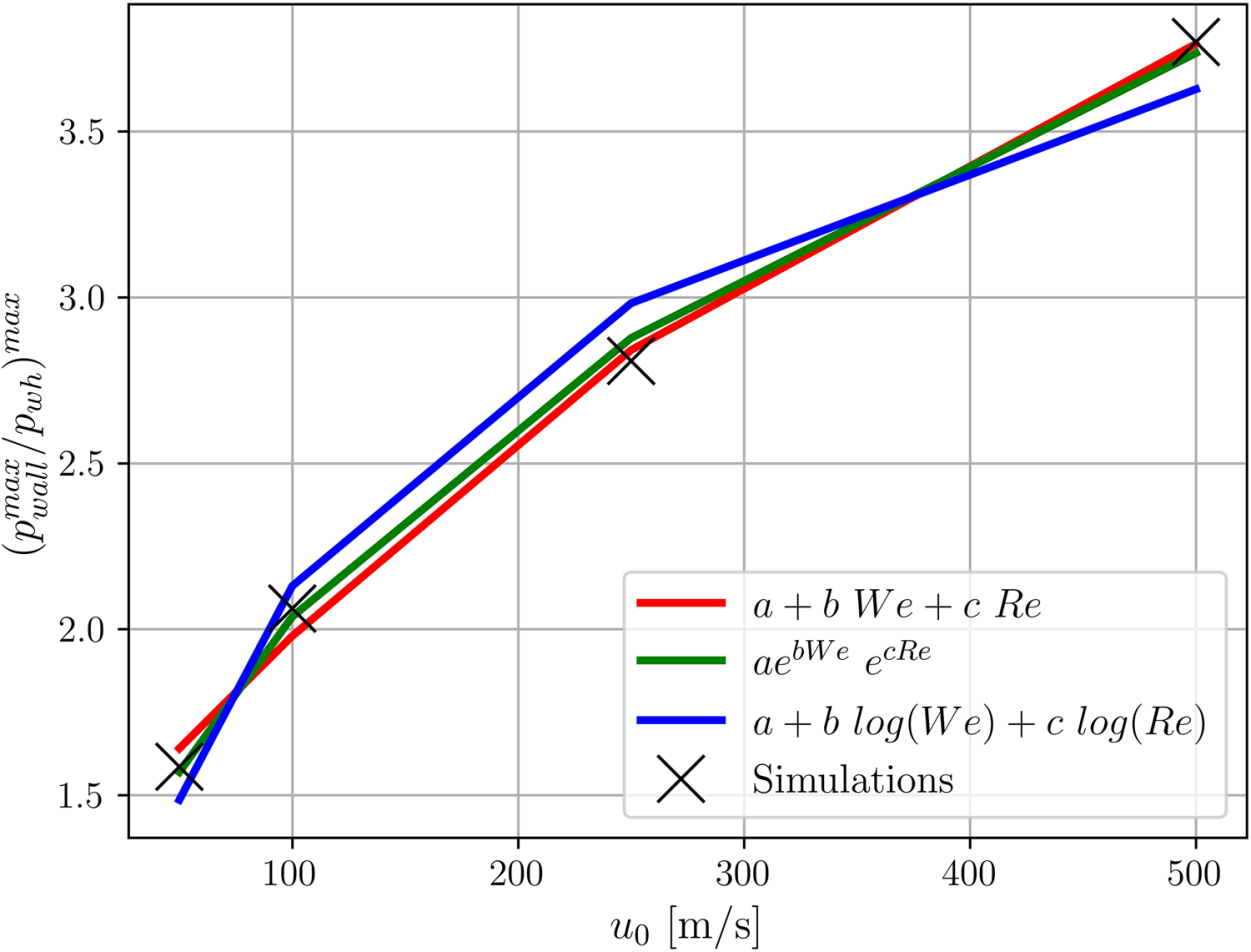
Different correlations proposed for maximum value of $p^{max}_{wall}$ observed during the evolution based on *We* and *Re*.

From application point of view the prediction of the pressure on the wall investigated in Fig. 6 is of interest. For example, the knowledge of the maximum pressure at the surface is important to prevent liquid impingement erosion, relevant in all the applications where a high speed droplet impacts onto a surface. Figure 7 suggests different correlations for the maximum value for normalised $p^{max}_{wall}$ as function of the *We* and *Re* numbers. Previous correlations to predict this value were based on the Mach number^4^. In our study, the correlations investigated are based on *We* and *Re* numbers, as commonly employed also to categorise the droplet impact. Among the correlations in Fig. 7 we find that the best fitting with the numerical data is given by Eq. (4).

4 $$\begin{aligned} \left( p_{wall}^{max}/p_{wh} \right) ^{max} = 155 \exp (0.81 We) \exp (-1.27 Re) \end{aligned}$$

**Figure 8:**
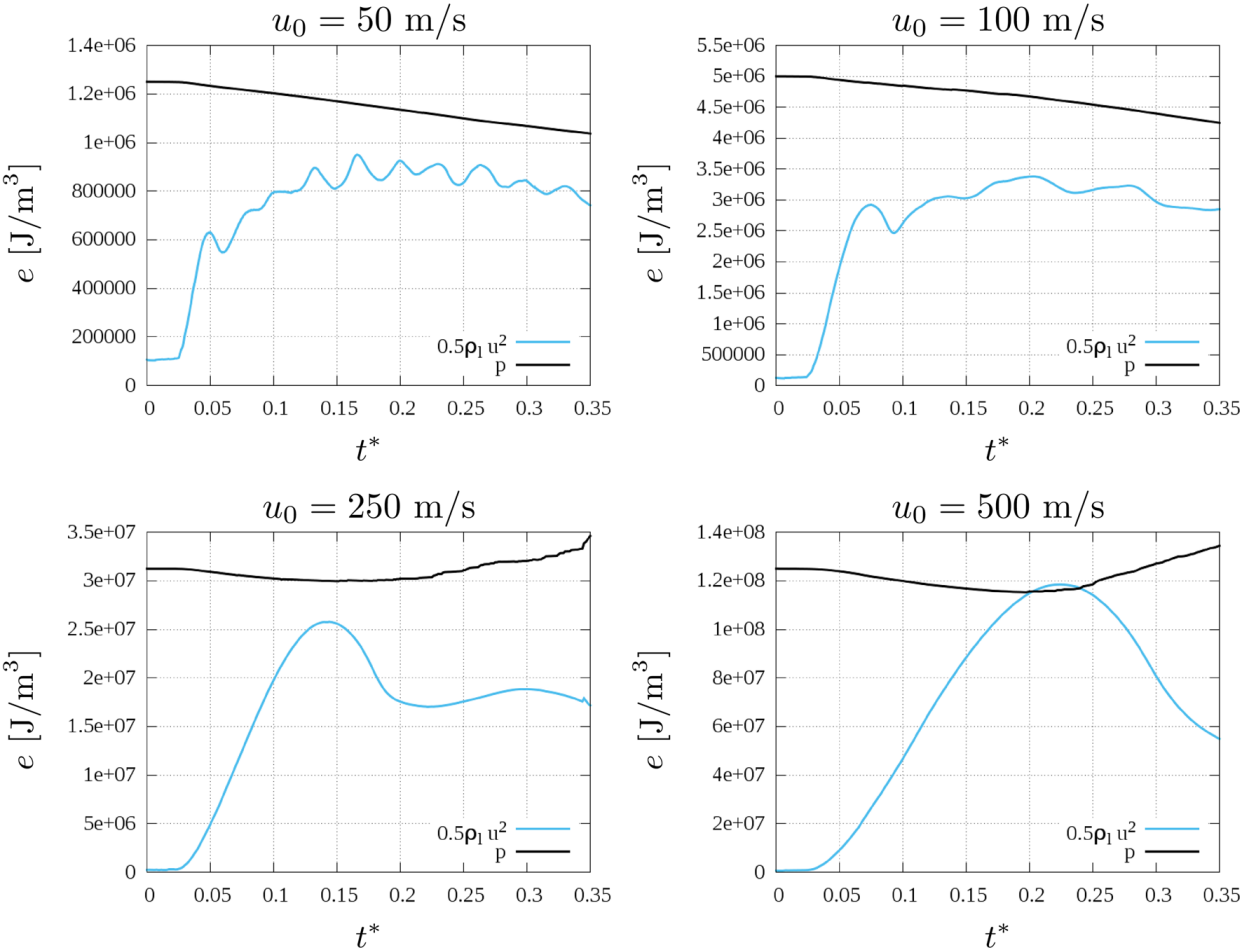
Pressure and Kinetic energy per unit of volume for the different velocities.

Another interesting aspect of the droplet pressure investigation is relevant to identifying how it relates to the kinetic energy $k_{d}$, in order to quantify the overall energy transformation during the impact. Figure 8 shows the trend over time of the pressure and kinetic energy per unit of volume over time. Both are calculated as droplet quantities, i.e. as done in Eq. (3). It is observed that while for the first two cases, the kinetic energy keeps decreasing and the pressure stabilises, increasing $u_{0}$, to a range that the compressibility within the droplet is not negligible ($u_{0} \ge 250 ~\text{ m/s }$) and once the maximum pressure $p_{d}$ is reached, the kinetic energy increases. When the compressibility within the liquid can not be neglected, the pressure peaks observed at the wall (Fig. 6a) propagate within the droplet, increasing its kinetic energy which otherwise will decrease after the impact.

### Surrounding gas dynamics

**Figure 9:**
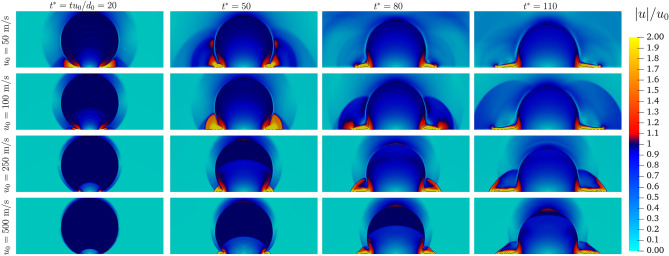
Velocity distribution around the droplet for different impact velocities at different $t^*$.

The influence of the initial impact velocity $u_{0}$ on the ambient surrounding of the droplet is now discussed. Figure 9 shows the trend of the velocity magnitude around the droplet, made dimensionless by the impact velocity $u_{0}$, for the different velocities at the same dimensionless instants $t^*$. From $u_{0} = 100 ~\text{ m/s }$ the splash jet creates a propagation front in the gaseous field. A further increase in the velocity results in a discontinuity front preceding the lamella advancement. The jet formed creates a propagation front in the gaseous field.

**Figure 10:**
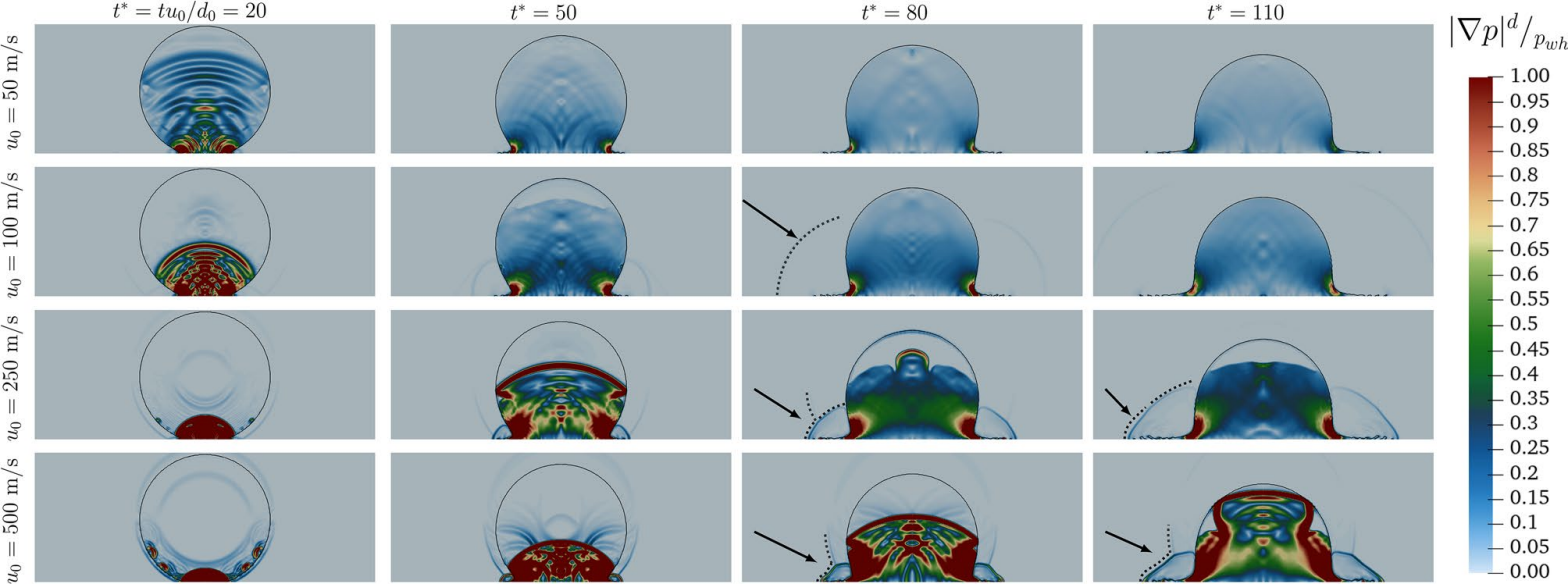
Dimensionless pressure gradient distribution around the droplet for different impact velocities at different $t^*$. The pressure gradient fronts are highlighted with dotted curved lines.

Complementary to Fig. 9, the discontinuity fronts can be highlighted by the pressure gradient in the surrounding gas in Fig. 10. The pressure gradient fronts are highlighted on the figure with dotted curved lines. The propagation front induced by the jet spreading is present for $u_{0}= 100~\text{ m/s }$ and above. These pressure fronts are less intensive than the ones within the droplet. For this case, the front is quickly dissipated. Increasing the impact velocity, two pressure fronts are observed in the gaseous field: one preceding the lamella and a second oblique one. This latter originates from the droplet’s side. For lower $u_{0}$ the lamella advancement overrides the propagation from the droplet interface, which instead survives for the higher velocity ($u_{0} = 500~\text{ m/s }$).

**Figure 11:**
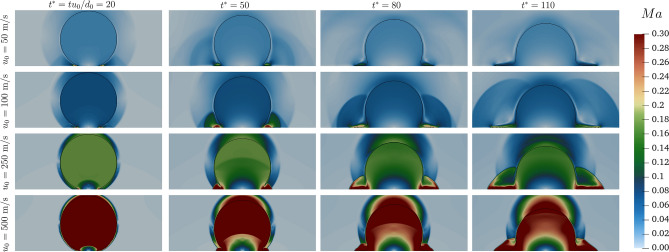
*Ma* distribution around the droplet for different impact velocities at different $t^*$.

As it becomes obvious from the previous observations the compressibility alters the dynamics inside and outside of the droplet. In order to highlight better this effect, Fig. 11 is included to visualise the distribution of the Mach number *Ma* inside the droplet. The upper limit of the observation range is set to 0.3, i.e. the limit of the incompressibility assumption. Since $u_{0}= 100~\text{ m/s }$, the impact point is characterised by $Ma \ge 0.3$. For $u_{0}= 100~\text{ m/s }$ the *Ma* decreases with time, while increasing $u_{0}$ further results in $Ma \ge 0.3$ on the whole lamella, with the discontinuity on the advancing region clearly visible. Only for $u_{0} = 500~\text{ m/s }$ the transition from incompressible to compressible regime is observed also within the droplet.

### Lamella dynamics

To investigate the lamella ejected, its velocity is calculated as

5 $$\begin{aligned} \varvec{u}_\ell = \frac{\int _{\Omega _\ell } \alpha \varvec{u} dx }{\int _{\Omega _\ell } \alpha dx } \end{aligned}$$

over the domain $\Omega _{\ell }$ surrounding the lamella.

**Figure 12:**
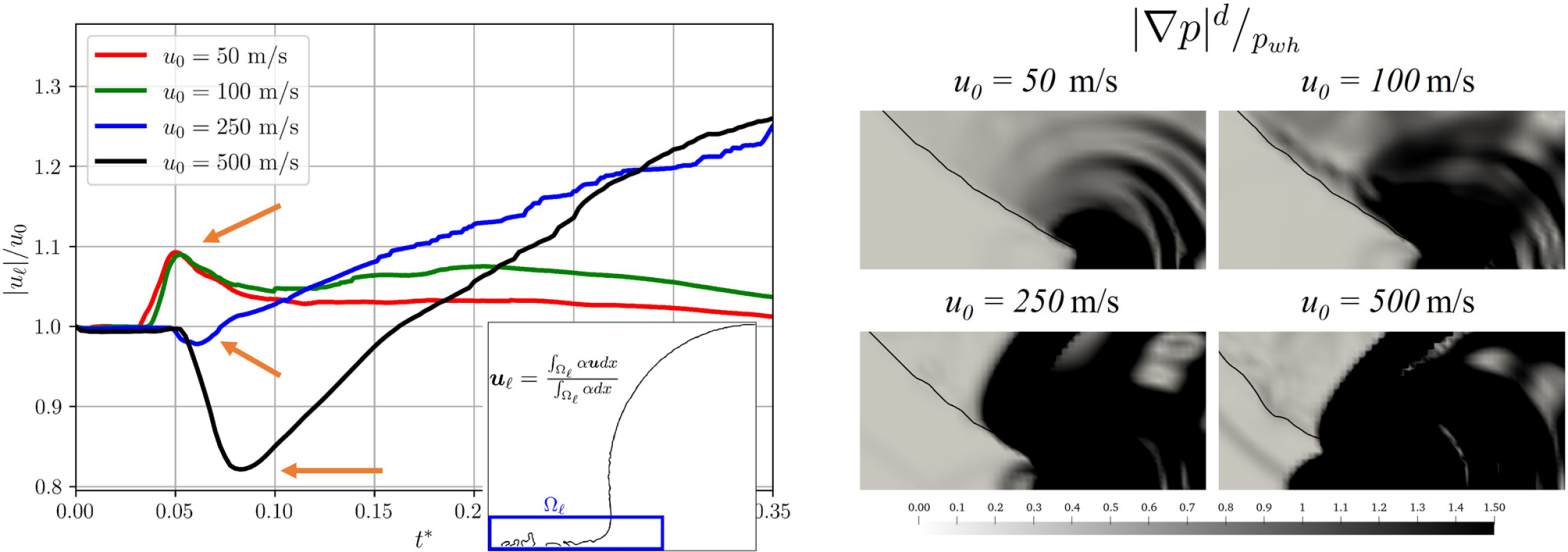
*Left:* trend over time of lamella speed $u_\ell$ for the different cases, with its schematisation. *Right:* pressure gradient distribution focused on the lamella ejection region. These snapshots are shown for each case at the same instants where the peaks for $|u|_\ell$ are observed (these are highlighted by the orange arrows in graph on the left).

Figure 12 shows on the left, the evolution of the lamella velocity magnitude $|\varvec{u}|_\ell$ for the different cases and on the right the pressure gradient distribution focused on the lamella ejection region. These snapshots are shown for each case at the same dimensionless instants where the peaks for $|u|_\ell$ are observed (these are highlighted by the orange arrows in graph on the left). For the cases with $u_{0} = 50$ and $100~\text{ m/s }$ the profiles are very similar: the velocity increases, reaching a maximum and then settles at a lower value, higher than the impact velocity. A further increase of $u_{0}$ changes the pattern observed: the velocity remains stable for a while, then starts decreasing, reaching a minimum value, and then increases linearly. For $u_{0} = 250~\text{ m/s }$ the minimum is close to $u_0$ and further decreases when $u_{0} = 500~\text{ m/s }$.

When $u_{0} \ge 250$, the lamella is affected by the front propagation of the pressure gradient, not observed for the lower case velocity. The presence of this pressure front crossing the lamella alters the velocity, with the reduction of $|u|_{\ell }$ that, after reaching the minimum increases. As observed in Fig. 11, for the two cases with higher $u_0$ the lamella shows $Ma \ge 0.3$, i.e. that region can not be considered incompressible. Thus, the compressibility of the liquid affects the velocity of the lamella, which for velocities up to $u_{0} = 100 ~\text{ m/s }$ is unaffected by the initial impact velocity, reaching the same $u_{\ell }/u_0$ peak at the same instants. On the other hand, when $u_{0} > 100 ~\text{ m/s }$ the trend is reversed, with a decrease of $u_\ell$ then followed by a linear increase in time.

## Conclusion

This study analyses the evolution of the impact of a liquid droplet hitting at high *We* and *Re* conditions a solid surface. Four different operating points have been investigated, varying the impact velocity from 50 to $500~\text{ m/s }$. This allows to observe how the impact dynamics change when compressibility of the droplet affects the phenomena. It should be noted that our investigation covers velocity ranges that until now have not been studied experimentally.

- Although no difference is observed in the droplet shape or normalised flow fields, increasing the impact velocity strengthens the role of the compressibility which in turn affects the travelling pressure propagation fronts within the droplet. For $u_0 \ge 250 ~\text{ m/s }$, a clear discontinuity is observed, that propagate from the impact point towards the top of the droplet in a direction normal to the wall. The waves are dissipated when reaching the interface, while are reflected for $u_{0} = 500~\text{ m/s }$.
- A further increase of the impact velocity induces secondary propagation fronts from the side. These interact with the main wave slowing its travel across the droplet.
- The maximum pressure at the wall increases with the increase of the impact velocity, exceeding $p_{wh}$ at the impact points. The peak values for the pressure are located at the corner of the impact, where the lamella is ejected, not in the centre. For all the cases, several pressure peaks, higher than the water hammer pressure $p_{wh}$, are observed in the earlier stage of the impact ($t^*\in [0.02:0.05]$). These pressure peaks increase with $u_{0}$.
- To predict pressure peaks experienced by the wall, we propose a correlation based on *We* and *Re* through Eq. (4), allowing to quantify the maximum stress at the wall.
- The ejection of the lamella alters the surrounding gaseous flow field. The lamella is ejected at high velocity ($> 2 u_0$), generating a main propagation wave within the gaseous field. A secondary wave is generated by the ejection of the lamella. These second waves are dissipated quickly for all the cases expect for $u_{0} = 500~\text{ m/s }$, where the wave interacts with the one generated by the lamella advancing resulting in a complex wave propagating into the air (see Fig. 10).
- Focusing on the *Ma* distribution, beyond $u_{0} = 250~\text{ m/s }$ the lamella region shows $Ma \ge 0.3$ during the whole impact, i.e. the incompressible assumption does not hold in the lamella, despite the rest of the droplet is still below this threshold.
- The compressibility present in the lamella region influences the lamella velocity $u_{\ell }$ (and thus the wetting behaviour of the droplet). When the lamella region shows $Ma < 0.3$, the $u_{\ell }$ profiles are not influenced by $u_{0}$. After the impact it reaches the same normalised maximum, then settles to a slightly lower value. When $Ma \ge 0.3$ is observed within the lamella (i.e. when $u_{0} \ge 250~\text{ m/s }$) the trend is different: after the impact, the lamella velocity is unaltered until it starts to decrease. After reaching a minimum value, a linear increase is observed. A further increase in $u_{0}$ reduces this minimum value.
- Although in this study the compressibility has been observed to be important for $u_0 \ge 250 ~\text{ m/s }$, it should be pointed out that the exact transition limit might depend also on other variables, such as the droplet diameter and the fluid properties. This aspect requires further investigation in future studies.

## Supplementary Information

Supplementary Information 1.

## Data Availability

The data that support the findings of this study are available from the corresponding author upon reasonable request.
