# Supplementary material for: Unveiling the dynamics of ultra high velocity droplet impact on solid surfaces
Source: Sci Rep. 2022 May 6;12:7416. doi: 10.1038/s41598-022-11188-7 (PMC9076664; doi:10.1038/s41598-022-11188-7)
Supplement: Supplementary file 1 — Supplementary Information 1. [file 41598_2022_11188_MOESM1_ESM.pdf]

1 Methodology

1.1 Governing Equations

Our suggested framework is designed for simulations of compressible and immiscible fluids. The inteface tracking of the two-phase flow is performed with the VoF method as basis using the Open Source code OpenFOAM[1]. The native solver *compressibleInterFOAM* was used as starting point and modifications were made to account for the interface treatment.

Being a one-fluid method, the continuity, energy and momentum equation for the mixture are solved:

$$\begin{aligned} &\frac{\partial \rho}{\partial t} + \nabla \cdot (\rho \boldsymbol{u}) = 0 \\ &\frac{\partial \rho \boldsymbol{u}}{\partial t} + \nabla \cdot (\rho \boldsymbol{u}\boldsymbol{u}) = \nabla \cdot (-p_d\, \boldsymbol{I} + \boldsymbol{\tau}) + \rho \boldsymbol{f}_b \\ &\frac{\partial \rho T}{\partial t} + \nabla \cdot (\rho \boldsymbol{u} T) = \nabla \cdot (k \nabla T) \end{aligned} \tag{1}$$

where $p_d = p - \rho \boldsymbol{g} \cdot \boldsymbol{x}$ is the peziometric pressure, f_b the body force and the mixture stress tensor $\boldsymbol{\tau}$ is defined as

$$\boldsymbol{\tau} = \mu \left(\nabla \boldsymbol{u} + \nabla \boldsymbol{u}^T - \frac{2}{3} \nabla \cdot \boldsymbol{u} \boldsymbol{I} \right) \tag{2}$$

To track the single phase, the transport equation for the volume fraction is solved

$$\frac{\partial \rho_1 \alpha}{\partial t} + \nabla \cdot (\rho_1 \alpha \boldsymbol{u}) = 0 \tag{3}$$

as only two phases are present, the volume fractions obey the algebraic relationship $\alpha_1 + \alpha_2 = 1$. The volume fraction is transported by the mixture velocity u.

When dealing with bounded scalars such as α, it is fundamental to respect the boundedness of the variable. For the α transport equation, Equation (3), the convective term can be source of unboundedness. Among the different strategies to mitigate the issue of the boundness, in this study the Multidimensional Universal Limiter with Explicit Solution (MULES), proposed by Weller[2], is adopted. The MULES limiter consists of an explicit solver which is based on the Flux Corrected Transport in order to maintain the boundedness of the scalar. A detailed description of the algorithm is beyond the scope of this study, and more details about its implementation can be found in literature[3–5]. Despite being derived in an incompressible framework, the MULES scheme can be directly applied to the compressible case. To be suitable to this scheme, the volume fraction equation is rearranged and the contribution from the compressibility is accounted numerically as a source term. This allows to hold a similar numerical methodology for the transport of the volume fraction between the compressible and incompressible solvers. The methodology presented is pressure-based, which means that the pressure is solved, with the phase densities obtained algebraically through the equation of state. The mixture density is defined as

$$\rho = \rho_1 \alpha + \rho_2 (1 - \alpha) \tag{4}$$

The transport properties of the mixture are calculated from the single phase properties through the volume fraction, for example the viscosity is defined by $\mu = \mu_1 \alpha + \mu_2 (1 - \alpha)$.

As the gravity force is included in the pressure gradient through p_d, the only body force present is the surface tension force. The surface tension force is treated as a pressure gradient across the liquid–gas interface and is calculated per unit volume based on the Continuum Surface Force (CSF) model proposed by Brackbill et al.[6].

$$\boldsymbol{f}_b = \sigma \kappa \boldsymbol{n}_I \tag{5}$$

where the mean interface curvature κ is defined as

$$\kappa = \nabla \cdot \boldsymbol{n}_I \tag{6}$$

and the normal to the interface $\boldsymbol{n}_I$ is defined as

$$\boldsymbol{n}_I = \frac{\nabla \alpha}{|\nabla \alpha|} \tag{7}$$

1.2 Balance Equation: non conservative form

When dealing with bounded scalar, it is fundamental to respect the boundedness of the variable. Referring to the α transport equation, (3), the convective term can be source of unboundedness.

Among the different strategies, in this study the Multidimensional Universal Limiter with Explicit Solution (MULES), proposed by Weller[2] and present in OpenFOAM, is adopted. The MULES limiter consists into an explicit solver which is based on the Flux Corrected Transport in order to maintain the boundedness of the scalar. To be suitable for the MULES limiter, the balance equation for the volume fraction is rearranged. Considering the mass balance for a generic phase k, the phase density is treated as a function of pressure: $\rho_k = \rho_k(p)$. Eq. (3), for phase k, is then rearranged as

$$\frac{\partial \alpha_k}{\partial t} + \boldsymbol{u}\cdot\nabla\alpha_k + \alpha_k\nabla\cdot\boldsymbol{u} + \frac{\alpha_k}{\rho_k}\left(\frac{\partial \rho_k}{\partial t} + \boldsymbol{u}\cdot\nabla\rho_k\right) = 0 \tag{8}$$

Applying the chain rule to the derivatives of density, it is obtained

$$\frac{\partial \alpha_k}{\partial t} + \boldsymbol{u}\cdot\nabla\alpha_k = -\alpha_k\nabla\cdot\boldsymbol{u} - \frac{\alpha_k}{\rho_k}\frac{\partial \rho_k}{\partial p}\left(\frac{\partial p}{\partial t} + \boldsymbol{u}\cdot\nabla p\right) \tag{9}$$

Summing Eq. (9) over both phases, it is obtained for the total mass

$$\left(\frac{\alpha_1}{\rho_1}\frac{\partial \rho_1}{\partial p} + \frac{\alpha_2}{\rho_2}\frac{\partial \rho_2}{\partial p}\right)\left(\frac{\partial p}{\partial t} + \boldsymbol{u}\cdot\nabla p\right) + \nabla\cdot\boldsymbol{u} = 0 \tag{10}$$

Replacing the right hand side term of Eq. (9) with Eq. (10), the final form for the transport of the balance equation is obtained

$$\frac{\partial \alpha}{\partial t} + \boldsymbol{u}\cdot\nabla\alpha = -\alpha\,(1-\alpha)\left(\frac{\rho_{1,p}\rho_1 - \rho_{2,p}\rho_2}{\alpha\rho_{1,p}\rho_2 + (1-\alpha)\,\rho_{2,p}\rho_1}\right)\nabla\cdot\boldsymbol{u} \tag{11}$$

where $\rho_{k,p} = \partial\rho_k/\partial p$.

2 Numerical Framework for the Interface Treatment

The numerical treatment of the interface properties and of the capillary force is presented in this section.

2.1 Discretisation of the Interface Curvature

After the value of the volume fraction at the cell centres is updated, the interface geometrical properties such as the mean curvature and its normal are calculated. From these properties the surface tension force for the momentum equation is then derived. To improve the accuracy of the the interface normal vectors near the interface, the volume fraction is subject initially to a smoothing process.

The smoothing process occurs in two stage: first the smoothed interpolating α from cell centres to face centres and then back to the cell centres recursively[7]. The smoothed volume fraction is then subject to a second cycle of smoothing, repeated two times, which consists into a weighted average of the volume fraction at the face with the face area. The smoothed volume fraction is then employed to calculate the interface normal vector n_I from Equation (7).

Once the interface normal vectors are computed, the interface curvature can be obtained from n_I through (6). The mean curvature calculated is smoothed in the direction normal to the interface recursively for two iterations ($i = \{0,1\}$), following Raeini et al.[7], as:

$$\kappa_{s,i+1} = 2\sqrt{\alpha\,(1-\alpha)}\,\kappa + \left(1 - 2\sqrt{\alpha\,(1-\alpha)}\right)\kappa_s^* \qquad , \kappa_{s,0} = \kappa \tag{12}$$

with

$$\kappa_s^* = \frac{\langle\langle \kappa_{s,i} w\rangle_{c\to f}\rangle_{f\to c}}{\langle\langle w\rangle_{c\to f}\rangle_{f\to c}} \qquad , w = \sqrt{\alpha\,(1-\alpha) + 10^{-6}} \tag{13}$$

The coefficient $2\sqrt{\alpha\,(1-\alpha)}$ diffuses κ away from the interface, with minimum effect of the values of κ on the interface region. This is important for the stability of the numerical method. Furthermore, the interface curvature at face centres (k_f) is obtained using a weighted interpolation method, as suggested by Renardy and Renardy[8]:

$$\kappa_f = \frac{\langle \kappa_{s,2} w\rangle}{\langle w\rangle} \qquad , w = \sqrt{\alpha\,(1-\alpha) + 10^{-6}} \tag{14}$$

2.2 Volume Fraction Sharpening and Capillary Forces

The capillary forces are calculated at the face centres, following Equation (15), as

$$f_{c,f} = \boldsymbol{f}_c \cdot \boldsymbol{n}_f = (\sigma \kappa \boldsymbol{n}_s \delta_s)_f \cdot \boldsymbol{n}_f = \sigma \kappa_f \delta_{s,f} \tag{15}$$

where $\boldsymbol{n}_f$ is the cell face normal, κ_f is the face value of the curvature, Equation (14). The interface delta function $\delta_{s,f}$ is calculated from the sharpened version of the volume fraction α_{sharp}, as $\delta_{s,f} = \nabla_f^{\perp}(\alpha_{pc})$, where $\nabla_f^{\perp}$ denotes the gradient normal to the face f. Using $\delta_{s,f}$ allows to control the sharpness of the capillary force, i.e., the thickness of the region where this force intervene.

The sharpened volume fraction α_{sharp}, following Raeini et al.[7], is obtained by curtailing and then normalising the volume fraction α as

$$\alpha_{sharp} = \frac{1}{1-C_{pc}}\left[\min\left(\max\left(\alpha, \frac{C_{pc}}{2}\right), 1-\frac{C_{pc}}{2}\right) - \frac{C_{pc}}{2}\right] \tag{16}$$

A value of $C_{pc} = 0$ lead to the original CSF (Continuum Surface Force) formulation (i.e., using α instead of α_{sharp} for the interface location), while as $C_{pc} \to 1$, α_{sharp} becomes sharper, leading to a sharper implementation of the capillary forces. For static cases a value of $C_{pc} = 0.98$ is suggested[7], while $C_{pc} = 0.5$ is used for the dynamic cases.

2.3 Filtering of the Capillary Forces

The adoption of $\delta_{s,f}$ allows to mitigate the non-physical velocities at the interface. When interaction with a boundary is present though, Raeini et al.[7] showed that this may not be enough, and non-physical currents close to complex (curved or edged) solid boundaries can still be present. This problem can be solved modifying those components of the capillary forces that result in currents parallel to fluid interfaces trough the following operator:

$$f_{c,f,filtered} = f_{c,f} - f_{c,f,filt||} \tag{17}$$

where $f_{c,f}$ is calculated through Equation (15), and $f_{c,f,filt||}$ is a time dependent term defined at the face centres.

Starting from zero as initial condition, this term is updated as:

$$f_{c,f,filt||} = \frac{\delta_{s,f}}{(\delta_{s,f}+\varepsilon)}\left(f^{old}_{c,f,filt||} + C_{c,f,filt||}\langle \nabla p_c - (\nabla p_c \cdot \boldsymbol{n}_s)\boldsymbol{n}_s \rangle_f \cdot \boldsymbol{n}_f\right) \tag{18}$$

where $f^{old}_{c,f,filt||}$ represents the value of $f_{c,f,filt||}$ at the previous time step.

The term $\frac{\delta_{s,f}}{(\delta_{s,f}+\varepsilon)}$ restricts the correction term to the region where the capillary force is present. Equation (18) gradually dampens those components of $f_c - \nabla p_c$ that are parallel to the interface, so that they finally converge to zero. This filtering may introduce small errors in the calculation, however the effect of this was observed to be negligible in the absence of non-smooth solid boundaries. The term $C_{c,f,filt||}$ is a coefficient determining how fast the non–physical velocities are filtered. A value of $C_{c,f,filt||} = 0.1$ is used in[7]

2.4 Filtering Capillary Fluxes

Due to the numerical errors in the calculation of the interface curvature, it is difficult to obtain a zero net capillary force constraint (i.e., $\oint \boldsymbol{f}_c \cdot \boldsymbol{S}_s = 0$, where $\boldsymbol{S}_s$ is the interface area vector) when modelling the movement of a closed interface. This issue is evident for configuration at low capillary numbers[7,9,10]. In this study, this strategy will still be investigated even if the applications are characterised by high Re and We, as the local capillary force where the interface is interacting with the boundary, may still play an important role in the process. Therefore, filtering the non-physical fluxes generated due to the inconsistent calculation of capillary forces is necessary to maintain the zero-net capillary force constraint.

A simple thresholding scheme to filter the capillary fluxes $\phi_c = |\boldsymbol{S}_f|\left(f_{c,f} - \nabla_f^{\perp} p_c\right)$ can be used[7] in order to enforce a zero net capillary force flux on closed interface. This filtering will explicitly set the capillary fluxes to zero when their magnitude is of the order of the numerical errors.

The filtered capillary flux is defined as

$$\phi_{c,filt} = \phi_c - \max\left(\min\left(\phi_c, \phi_{c,thr}\right), -\phi_{c,thr}\right) \tag{19}$$

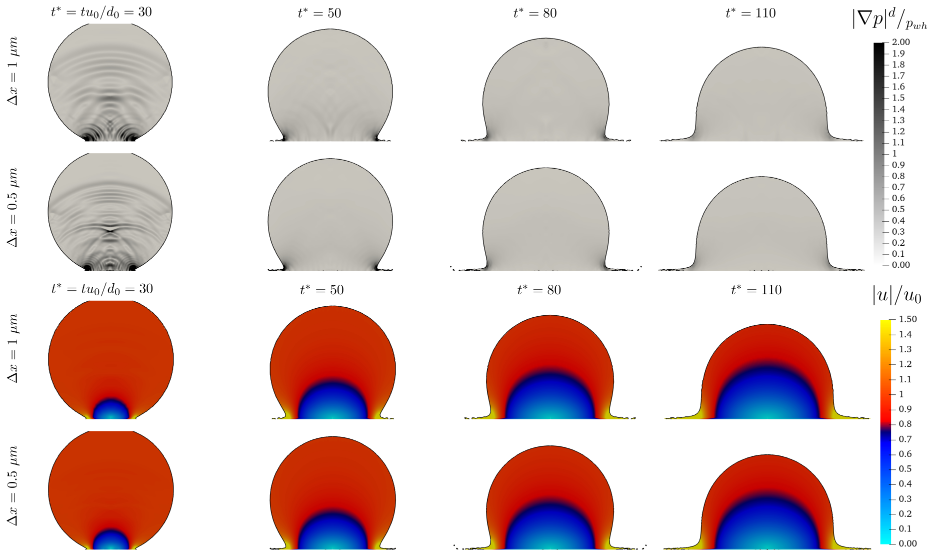

Figure 1. Distribution, at different times, of (*top*) dimensionless pressure gradient distribution and (*bottom*) dimensionless velocity for both the cell sizes $\Delta x = 1\ \mu$m and $\Delta x = 0.5\ \mu$m.

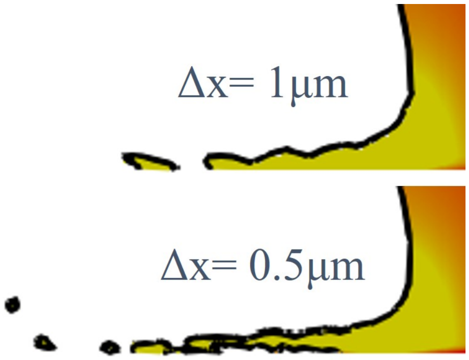

Figure 2. Zoom of the lamella ejected changing the cell sizes.

where $\phi_{c,thr}$ is a threshold value below which capillary fluxes are set to zero. The threshold value is chosen as

$$\phi_{c,thr} = C_{\phi c,thr} |f_{c,f}|_{avg} |\boldsymbol{S}_f| \tag{20}$$

where $|f_{c,f}|_{avg}$ is the average value of capillary forces (Equation (15)) over all faces where $f_{c,v} \neq 0$. The coefficient $C_{\phi c,thr}$ define the threshold of the capillary fluxes to be filtered. This threshold indicates that the fluxes that are within this range of numerical error are filtered. For example, a value of $C_{\phi c,thr} = 0.01$ implies that the capillary fluxes are set to zero if their magnitude is less than 1% of the average of the capillary forces. The aim of this filtering is to prevent the capillary forces to cause instabilities or to introduce large errors in the velocity fields.

3 Grid Dependency

To investigate the influence of the grid, the case with $u_0 = 50$ m/s is simulated with two different meshes. The first with a cell size of $\Delta x = 1\mu$m ($d/n_c = 200$) and the second with a grid size of $\Delta x = 0.5\mu$m ($d/n_c = 400$). The spreading, maximum pressure at the wall and the flow field distributions are compared.

Figure 1 shows the distribution within the droplet, at different times, for both the dimensionless pressure gradient and velocity changing the grid size. No sensible difference is observed increasing the resolution for both the fields.

In Fig. 2 the focus is on the lamella ejected and how it changes reducing the grid size. As expected, reducing the grid size the droplet produced from the lamella can be detected. Apart from this, the lamella shape and length is not affected by the resolution. As the topic of this study is not on the secondary droplet produced, the morphology of the lamella is correctly predicted also with $\Delta x = 1\mu$m.

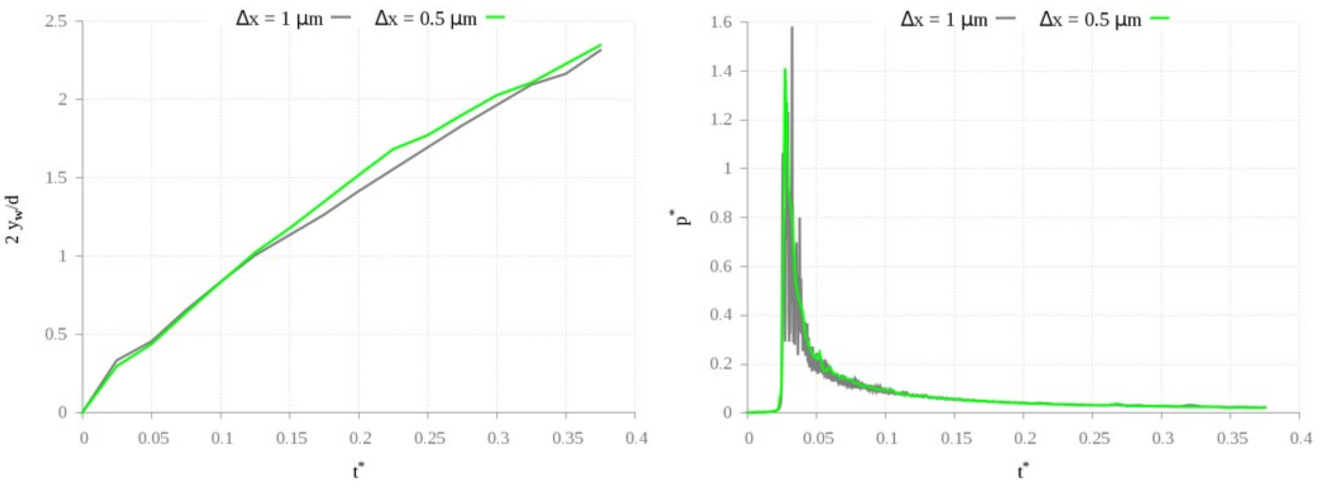

(a) Spreading   **(b)** p_{wall}^{max}

Figure 3. Droplet spreading and maximum pressure at the wall during the impact changing the grid size.

A quantitative comparison is offered in Fig. 3, which shows the spreading and maximum pressure at the wall during the impact changing the grid size. Both the quantities are not affected by the resolutions investigated.

Thus, a grid size of $\Delta x = 1\mu$m is employed for the rest of the study.

References

1. OpenCFD. *OpenFOAM - The Open Source CFD Toolbox - User's Guide*. OpenCFD Ltd., United Kingdom (2018).
2. Weller, H. G. A new approach to VOF-based interface capturing methods for incompressible and compressible flow. *OpenCFD Ltd., Rep. TR/HGW/04* (2008).
3. Damián, S. M. *An Extended Mixture Model for the Simultaneous Treatment of Short and Long Scale Interfaces*. Ph.D. thesis, Universidad Nacional del Litoral (2013). DOI: 10.13140/RG.2.1.3182.8320.
4. Tretola, G. *Large Eddy Simulation of atomisation process using the Eulerian Stochastic Fields method*. Ph.D. thesis, Imperial College London (2021).
5. Tretola, G., Vogiatzaki, K. & Navarro-Martinez, S. Implementation of a probabilistic surface density volume of fluid approach for spray atomisation. *Comput. & Fluids* 105121, DOI: https://doi.org/10.1016/j.compfluid.2021.105121 (2021).
6. Brackbill, J. U., Kothe, D. B. & Zemach, C. A continuum method for modeling surface tension. *J. computational physics* **100**, 335–354 (1992).
7. Raeini, A. Q., Blunt, M. J. & Bijeljic, B. Modelling two-phase flow in porous media at the pore scale using the volume-of-fluid method. *J. Comput. Phys.* **231**, 5653 – 5668, DOI: https://doi.org/10.1016/j.jcp.2012.04.011 (2012).
8. Renardy, Y. & Renardy, M. Prost: a parabolic reconstruction of surface tension for the volume-of-fluid method. *J. computational physics* **183**, 400–421 (2002).
9. Abadie, T., Aubin, J. & Legendre, D. On the combined effects of surface tension force calculation and interface advection on spurious currents within Volume of Fluid and Level Set frameworks. *J. Comput. Phys.* **297**, 611–636, DOI: 10.1016/j.jcp.2015.04.054 (2015).
10. Aboukhedr, M., Georgoulas, A., Marengo, M., Gavaises, M. & Vogiatzaki, K. Simulation of micro-flow dynamics at low capillary numbers using adaptive interface compression. *Comput. Fluids* **165**, 13–32, DOI: 10.1016/j.compfluid.2018.01.009 (2018).
